# Metabolic alterations of endothelial cells under transient and persistent hypoxia: study using a 3D microvessels-on-chip model

**DOI:** 10.1080/21688370.2024.2431416

**Published:** 2024-11-25

**Authors:** Kanchana Pandian, Anton Jan van Zonneveld, Amy Harms, Thomas Hankemeier

**Affiliations:** a Division of Systems Biomedicine and Pharmacology, LACDR, Leiden University, Leiden, The Netherlands; b Department of Internal Medicine (Nephrology) and the Einthoven Laboratory for Vascular and Regenerative Medicine, Leiden University Medical Center (LUMC), Leiden, The Netherlands

**Keywords:** 3D microvessels-on-chip model, endothelial dysfunction, hypoxia, hypoxia inducible factor – 1 alpha, nitric oxide metabolites, signaling lipids

## Abstract

Numerous signaling pathways are activated during hypoxia to facilitate angiogenesis, promoting interactions among endothelial cells and initiating downstream signaling cascades. Although the pivotal role of the nitric oxide (NO) response pathway is well-established, the involvement of arginine-specific metabolism and bioactive lipid mechanisms in 3D flow-activated in vitro models remains less understood. In this study, we explored the levels of arginine-specific metabolites and bioactive lipids in human coronary artery endothelial cells (HCAECs) under both transient and persistent hypoxia. We compared targeted metabolite levels between a 2D static culture model and a 3D microvessels-on-chip model. Notably, we observed robust regulation of NO metabolites in both transient and persistent hypoxic conditions. In the 2D model under transient hypoxia, metabolic readouts of bioactive lipids revealed increased oxidative stress markers, a phenomenon not observed in the 3D microvessels. Furthermore, we made a novel discovery that the responses of bioactive lipids were regulated by hypoxia inducible factor-1α (HIF-1α) in the 2D cell culture model and partially by HIF-1α and flow-induced shear stress in the 3D microvessels. Immunostaining confirmed the HIF-1α-induced regulation under both hypoxic conditions. Real-time oxygen measurements in the 3D microvessels using an oxygen probe validated that oxygen levels were maintained in the 3D model. Overall, our findings underscore the critical regulatory roles of HIF-1α and shear stress in NO metabolites and bioactive lipids in both 2D and 3D cell culture models.

## 1. Introduction

The vascular endothelium, the first cellular layer in contact with blood, continuously adapts to a variety of physiological and pathological environmental cues to maintain homeostasis. One challenging stimulus is an oxygen tension deficit.^1^ While the response of the endothelial cells (ECs) to hypoxic stress can differ depending on the degree and duration of the hypoxia and organ specific vascular bed, it mostly entails the modulation of vasodilation, constriction, and cell proliferation through transcriptional responses driven by HIFs.^2^ Additional physiological factors, like blood flow and shear stress may further modulate the hypoxic endothelium-based homeostatic mechanisms. Two central signaling pathways involved in the endothelial response to hypoxia involve the endothelial nitric oxide synthase (eNOS) pathway^3–6^ and mechanisms involving the signaling lipids.^7^

In the presence of sufficient co-factors, eNOS can catalyze the formation of the potent vasodilator nitric oxide (NO) from oxygen and L-arginine.^8–10^ However, in patients at risk for cardiovascular disease, systemic low grade inflammatory conditions and tissue ischemia, this enzyme can become ‘uncoupled’ leading to the leak of electrons from the transport chain in the reductase domain to molecular oxygen. As a consequence, the superoxide anion (O^−^) is formed that can react with residual NO to yield peroxynitrite (ONOO^−^).^11–13^ Recently, NO was found to attenuate the accumulation of HIF-1α under hypoxic conditions through various mechanisms. These mechanisms include the redistribution of oxygen toward non-respiratory oxygen-dependent targets like HIF-1α proline hydroxylases(PHDs), which perform hydroxylation of Pro402/564 of HIF-1α leading to its proteasomal degradation, as well as the formation of peroxynitrite, which increases cytosolic iron/2-oxoglutarate levels required for PHD activation. Here, we propose a hypothesis that peroxynitrite, formed in the cells upon exposure to NO under low oxygen availability, serves as an alternative donor of oxygen for activated PHDs so they can perform HIF-1α proline hydroxylation to de-accumulate the protein.^14^ In addition, any disturbance of this complex regulatory mechanism causes changes to NO mechanisms, leading to increased cellular oxidative stress and decreased NO bioavailability. Hypoxic conditions and the downregulation of eNOS have been studied widely in different cell models under hypoxic conditions such as in HUVECs,^15,16^ in HCAECs,^17^ in porcine aortic endothelial cells (PAECs),^18^ as well as in vivo conditions such as pulmonary hypertension patients^19^ and aortic and mesenteric arteries of mice exposed to chronic intermittent hypoxia.^20^ In contrast, some studies have reported the upregulation of eNOS in hypoxia induced persistent pulmonary hypertension of the newborn rat,^21^ and in vitro cultured PAECs also doubled the nitrite level after exposed to hypoxia.^22^

Similarly, endothelial adaptations to hypoxia involve alterations in phospholipase A2 (PLA2) metabolism, a system that generates bioactive lipids that can mediate in a variety of signaling cascades including vascular homeostasis and inflammation.^23–25^ Hypoxia is associated with higher expression of lipoxygenases (LOX)^26^ and cyclooxygenase-2 (COX-2), the inducible cyclooxygenase in most tissues.^27–30^ Typical signaling lipid classes from arachidonic acid (AA) metabolism involve oxylipins and (nitro) free fatty acids, and lysophospholipids. In the vascular endothelium, hypoxia elicits an inflammatory response^31,32^ characterized by an elevated production of reactive oxygen species (ROS) leading to stabilization of HIF-1α.^33^ These ROS effects in ECs were investigated with assessment of stable ROS marker metabolites in lipid peroxidation and went hand in hand with reduced NO production.^15,17,34–37^

In this study, we demonstrated the transient and persistent hypoxic response of endothelial cells in 2D steady culture and 3D microvessels model in terms of metabolic standpoint. Using stable isotopes, we tracked the eNOS pathway by using isotope labeled L-arginine, the NO substrate, to track its downstream metabolites L-Citrulline and L-Ornithine. The findings reveal no statistically significant differences in the levels of L-citrulline and L-ornithine when comparing normoxia to hypoxia in both 2D and 3D culture models. In addition, we analyzed the signaling cascades of bioactive lipids under transient and persistent hypoxia. In the 2D model under transient hypoxia, the metabolic readout of bioactive lipids demonstrated an increase in oxidative stress markers, a trend not observed in 3D microvessels. Under persistent hypoxia, both 2D and 3D models exhibited downregulation of bioactive lipids associated with inflammation. Furthermore, immunostaining confirmed the higher nuclear localization of the hypoxia marker HIF-1α in persistent hypoxia.

## 2. Materials and methods

### 2.1. Cell culture models and oxygen treatment

HCAECs (PromoCell, C-12221) were resuspended in 10 ml fresh EGM MV2 medium with supplements (PromoCell, C-22022, C-39216) and cultured in T75 flasks (Nunc Easyflask, Sigma, F7552). Cell cultures were maintained at 37°C with 5% CO_2_ and media was refreshed three times a week. Cells were detached at 85% confluence with 0.25% Trypsin EDTA (Lonza, CC-5012) and cell pellets were collected by centrifugation at 300 g for 5 minutes.

For 2D culture experiments, the collected cell pellets were suspended in a fresh medium to a concentration of 7 × 10^5^ cells/ml and cultured in a 48 well plate for 48 hours. To synchronize or equilibrate the cell cycle we treated cells with Krebs buffer solution, pH 7.4 (Thermo fisher Scientific, J67795.AP) for 1 hour before the start of each experiment. Subsequently the cells were incubated for 12 and 48 hours with 100 µL of RPMI SILAC (ThermoFisher Scientific 88,365) supplemented with 150 µM^13^C_6_, ^15^N_4_-L-arginine (CORTECNET 130,541), and 2% FBS (ThermoFisher Scientific, A4736301) under 21% and 1% oxygen, balanced to 5% CO_2_ +74% N_2_ and 5% CO_2_ +94% N_2_ (Panasonic oxygen incubator).

For 3D cultures, we used a modified chip design 2-lane rerouted OrganoPlate (MIMETAS, Netherlands) that was designed to attach a microfluidic pump to apply fluid flow and was adapted from the work described in.^38^ The protocol for the 3D model is as follows: HCAECs were seeded into an OrganoPlate that was specially designed for controlled perfusion using a pump attachment. The cells were cultured for 3–4 days with media refreshed every other day. The detailed design and culture method of 3D microvessels model is described in Supplementary Material 1. For controlled flow of the media in the plate, a small motor is operated with the help of LabVIEW solutions software. This software allows the control of fluid flow rate and related shear stress (dyne/cm^2^), use of portal connections, motor position and value.Using the pump, the flow was operated to create shear stress of 5 dyne/cm^2^ and the cells were incubated under normoxia or hypoxia for 12 or 48 hours. The incubation protocols, and oxygen exposure were similar to that of the 2D assay described above.

After the incubation hours, the medium samples were collected separately in 1.5 mL Eppendorf tubes, snap frozen in liquid nitrogen and stored at −80${\;}^{\circ }$C for liquid chromatography and mass spectrometry (LCMS) analysis.

### 2.2. Metabolic profiling

For eNOS-based NO marker metabolite’s measurement, 20 µL of media were transferred to fresh Eppendorf tubes to equalize the sample volumes for further processes. Sample preparation procedure and LC-MS acquisition method were adapted from the previous work.^38^ Briefly, samples were analyzed using a targeted UPLC-MS/MS method that employed an AccQ-Tag derivatization strategy (Waters, Waters B.V. Art. No. 186003836, The Netherlands). Quality control (QC) samples were generated by pooling equal volumes of all cell media and cell lysate samples, and 10 µL of this pool underwent the same process as individual samples and QCs were injected every 10 study samples. For the MS measurements, 3 µl of sample solution was injected onto a UPLC Class I (Acquity, Waters Chromatography Europe BV, Etten-Leur, The Netherlands) system with an AccQ-Tag Ultra C18 Column (1.7 µm, 100 × 2.1 mm, Waters, Ireland) coupled to a Sciex QTRAP® 6500 mass spectrometer. For liquid chromatography (LC) separation, mobile phase A consisted of 0.1% formic acid in water. Mobile phase B consisted of 0.1% formic acid in acetonitrile. The final flow rate used was 0.7 mL/min and the end gradient was 99.8% A and 0.2% B mobile phase. The ESI source parameters were same as previous work. Data acquisition was performed in multiple reaction monitoring mode targeting compounds with different labeling statuses. The compound list with target m/z for parent and product ions is shown in Supplementary Table 1. Raw LC-MS/MS data was processed with AB Sciex PeakView™ 2.0 and MultiQuant™ 3.0.1 software for targeted metabolite peak identification and integration. Metabolite isotopologue levels were quantified using the corresponding peak areas.

For signaling lipids measurements, metabolite extraction, sample preparation procedure was according to the in-house experimental protocol which has been standardized and published.^39^ Briefly, the cell media samples were thawed on ice, and randomized prior to analysis. The sample media volume of 50 µL for each condition was aliquoted in fresh tubes and the remains were pooled and used for quality control (QC) samples. The thawed samples underwent the addition of 2.5 µL of antioxidant solution and 5 µL of internal standards (ISTDs). Acidification with citric acid/phosphate buffer (pH 4.5) followed, and all samples underwent liquid-liquid extraction (LLE) using 1 mL of butanol and ethyl acetate (1:1 v/v). Following vortexing and centrifugation, the organic phase was collected and subjected to drying. After reconstitution with an ice-cold 70% MeOH injection solution, each sample underwent additional vortexing and centrifugation, and the resulting supernatant was transferred to the insert in a glass vial.

The method makes use of two optimized chromatographic separations with different pH gradients. The first one is the High-pH runs, for that the injection volume of 5 µL was measured by a LCMS-8060 system (Shimadzu, Japan) with a Kinetex® Core-Shell EVO 100 Å C18 Column (50 × 2.1 mm, 1.7 µm) maintained at 40°C The MS system used was a Shimadzu LCMS-8060 triple-quadrupole mass spectrometer with an electrospray ionization source.

The chromatographic separations was achieved using two sample injection into two complimentary separations with different stationary phases and different gradients. We tailored a high pH run and low pH run for the classes of signaling lipids.^39^ For the low-pH runs, 5 µL was injected and analyzed using a LCMS system of Shimadzu LC-30AD hyphenated to a SCIEX Triple-Quad 7500 system with an electrospray ionization source (USA) with a Waters Acquity® BEH C18 column (50 mm × 2.1 mm, 1.7 µm) maintained at 40°C. QC samples and blank samples were injected together with study samples to ensure data quality. Metabolites showing a relative standard deviation (RSD) of no more than 30% on corrected peak areas in QC samples were used as a criterion for further analysis.

### 2.3. HIF-1α measurement

Cells from 2D culture model and 3D microvessels were fixed with 4% paraformaldehyde and 0.3% Triton X-100, washed with PBS, blocked with 0.5% Bovine Serum Albumin (BSA) for 30 min at room temperature, and stained with HIF-1⍺ antibody (#610,959; BD Biosciences, Franklin Lakes, NJ, USA) and Rhodamine Phalloidin (R415; Thermo Fisher Scientific, Waltham, MA, USA) overnight at 4°C and 2 h at RT, respectively. Cells were rinsed with 0.5% BSA every 10 min 3 times and stained with secondary antibody and Hoechst 33,258 (#610959; Thermo Fisher) for 1 h at room temperature (RT) in the dark. After washing with PBS, plates were stored at 4°C. Images were taken with a Nikon Eclipse Ti microscope with a 20 X objective. Scale bars shown in the images represent 50 μm.

### 2.4. Measurement of real time oxygen in a 3D microvessels-on-a-chip model

The cells were seeded into the 3D microvessels – OrganoPlate (similar protocol mentioned in the above section) which is specially designed for the pump attachment for unidirectional flow and in-line oxygen measurement.^38,40^ The pump is made of stainless steel and the tube connections for the fluid transfer from the inlet and outlet of the chips are made of silicone. For controlled rotation of the pump, a small motor is operated with the help of LabVIEW software.

For oxygen measurement in the 3D rerouted plate fluid flow conditions, we used the optical fiber-based detection system. The oxnano probe (Pyroscience, OXNANO oxygen nanoprobes) in cell growth media was infused onto a chip and calibrated the initial oxygen value in the oxygen incubator. We performed a two-point calibration with oxnano probe prepared in cell media and set to 21% oxygen and with sodium nitrite solution, which acts as an oxygen scavenger, and calibrated to 0% oxygen. The probe’s fluorescence intensities at various time points were used to determine the continuous measurement of oxygen within the integrated microvessels-on-chips (Supplementary Figure S1).

### 2.5. Statistical analysis

Bar plots were created with GraphPad Prism 9.3.1 software. Significance was determined by ANOVA, Tukey’s analysis, and student t-test. Lipid profiles – Volcano plots were plotted using R Studio version 2023.06.1.

## 3. Results

### 3.1. HCAECs respond to persistent oxygen exposure by increasing HIF-1α expression and nuclear relocation

**Figure 1.**
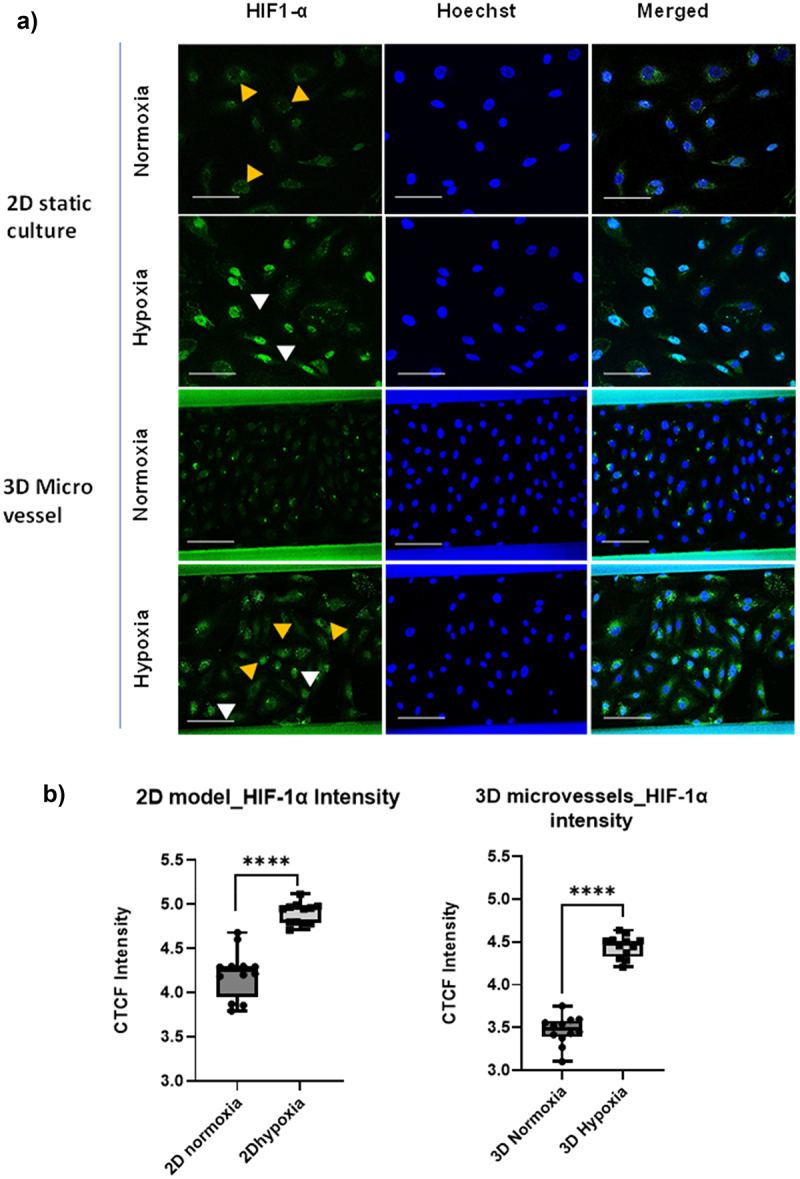
Immunofluorescence staining of HIF1- α. (a) HIF1- α localization by confocal microscopy after 48hrs (persistent) incubation under normoxia and hypoxia in 2D culture well plate and 3D microvessels-on-chip model. Green, HIF1- α Ab; blue, Hoechst; (biological experiments *n* = 2). White mark – localization of HIF1- α in the nucleus and brown arrow – No localization of HIF1- α in the nucleus. Scale bar −50 µm. (b) Fluorescence intensity of immunofluorescent cells calculated by corrected total cell fluorescence (CTCF) in 2D culture and 3D microvessels using ImageJ. Significance was calculated by a student T-test. ****p < 0.0001.

To validate the impact of hypoxia (1% oxygen) in our models (Supplementary Figure S2) we analyzed the expression of the key hypoxic biomarker HIF-1α by immunostaining. Compared to normoxic conditions, or short term 18 hour hypoxia (not shown), 48 hr persistent hypoxia led to a marked accumulation of HIF-1α in the nuclei of HCAECs in static 2D cultures (Figure 1(a,b)). Likewise, in the 3D HCAECs cultures, increased expression, and accumulation of HIF-1α a was observed in 50% of cell populations. Quantification of the fluorescent intensity of HIF-1α staining in both models showed a slightly higher HIF-1α level in the nuclei of the 2D cultured cells compared to those cultured in the 3D microvessels (Figure 1(a,b)).

### 3.2. Measurement of arginine-based NO metabolites in response to transient and persistent hypoxia exposure in HCAECs

**Figure 2.**
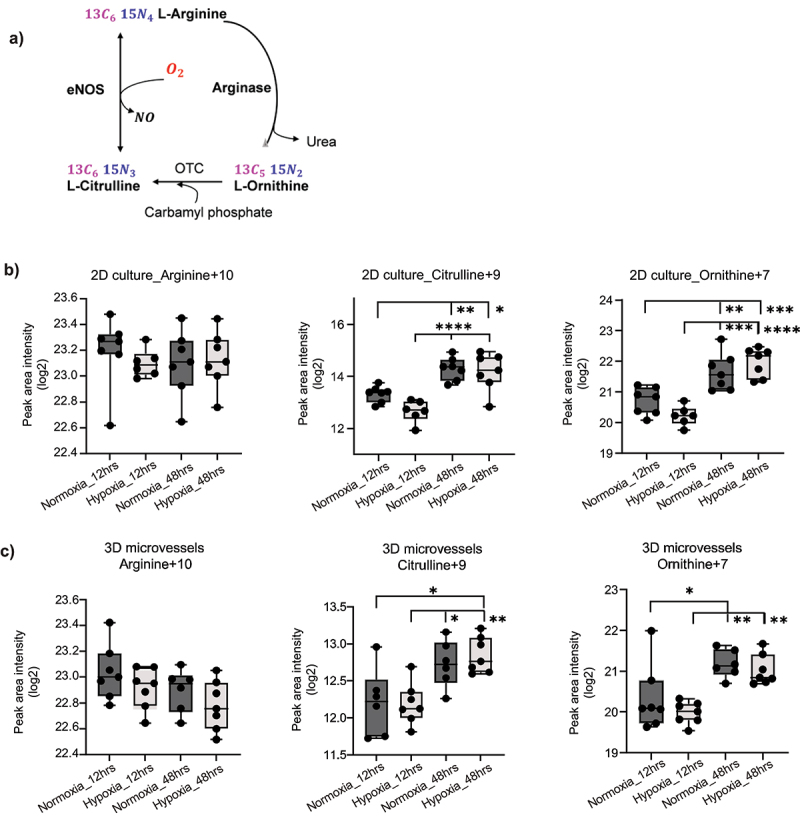
Measurement of isotope-labeled nitric oxide (NO) metabolites. (a) Metabolic pathway of ^13^C_6_^15^N_4_ L-arginine to ^13^C_6_, ^15^N_3_ L-Citrulline and ^13^C_5_, ^15^N_2_ L-Ornithine. Measurement of Arginine + 10 (^13^C_6_^15^N_4_ L-arginine), Citrulline + 9 (^13^C_6_, ^15^N_3_ L-Citrulline), and Ornithine + 7 (^13^C_5_, ^15^N_2_ L-Ornithine), (b) in 2D static culture, and (c) 3D microvessels-on-chip model under transient and persistent hypoxic conditions, *n* = 7. Significance is determined by the one-way ANOVA multiple comparison test. ns = not significant; *p < 0.05; **p < 0.01; ***p < 0.001; ****p < 0.0001.

In the presence of sufficient co-factors such as NADPH, FMAD, BH4, Ca2+, eNOS utilizes L-arginine and molecular oxygen to generate NO.^8,9,41^ In the process, L-arginine is converted to L-citrulline and therefore the rate of this conversion is widely used as a measure for eNOS activity (Figure 2(a)). Hence, to compare the impact of transient (12 hr) and persistent (48 hr) hypoxia on the activity of eNOS in our static 2D and 3D model systems, we measured the conversion of the NO substrate ^13^C_6_, ^15^N_4_ L-arginine to ^13^C_6_, ^15^N_3_ L-Citrulline by the enzyme. In addition, we assessed the generation of ^13^C_5_, ^15^N_2_ L-Ornithine by the arginase enzyme that also uses L-arginine as a substrate. HCAECs were cultured in 48-well cell culture plate and in our 3D microvessels-on-chip model that includes perfusion facilitated by a microfluidic pump.^38^ Figure 2(b,c) shows that while the L-arginine +10 levels are maintained, eNOS and arginase are active in both models, showing markedly elevated levels of L-citrulline +9 and L-ornithine +7 in conditioned media of both models after 48 hrs of hypoxia compared to the levels of these compounds measured after 12 hrs. However, no significant differences in citrulline + 9 and ornithine + 7 levels were observed between normoxia and hypoxia in the models.

When we compared the ratios of these target metabolites, the 2D static cultures exhibit no statistical significance in ratios such as citrulline + 9/ornithine +7, reflecting relative eNOS to arginase activity and ornithine + 7/arginine +10, indicating unchanged arginine utilization by arginase in both transient and persistent hypoxic conditions (Supplementary Figure S3). A minor lowering of the citrulline + 9/arginine +10 ratio was observed in transient 12 hr hypoxia when compared to normoxia in 2D static culture which indicated a decrease in the utilization of arginine by eNOS for NO production, but this difference was lost in the 48 hr in persistent hypoxia cultures.

In the 3D models, no significant differences between normoxia and hypoxia in the ratios could be observed. Interestingly, unlike the 2D model, there is a significantly lower level of citrulline + 9/ornithine +7 in both normoxia and hypoxia in microvessels exposed to persistent hypoxia, indicating that relative eNOS to arginase activity is lower and suggesting that the flow-mediated mechanical response may impact the hypoxic response of the HCAECs.

### 3.3. Generation of signaling lipids in transient and persistent hypoxic conditions in 2D static and 3D microvessels-on-chip model

**Figure 3.**
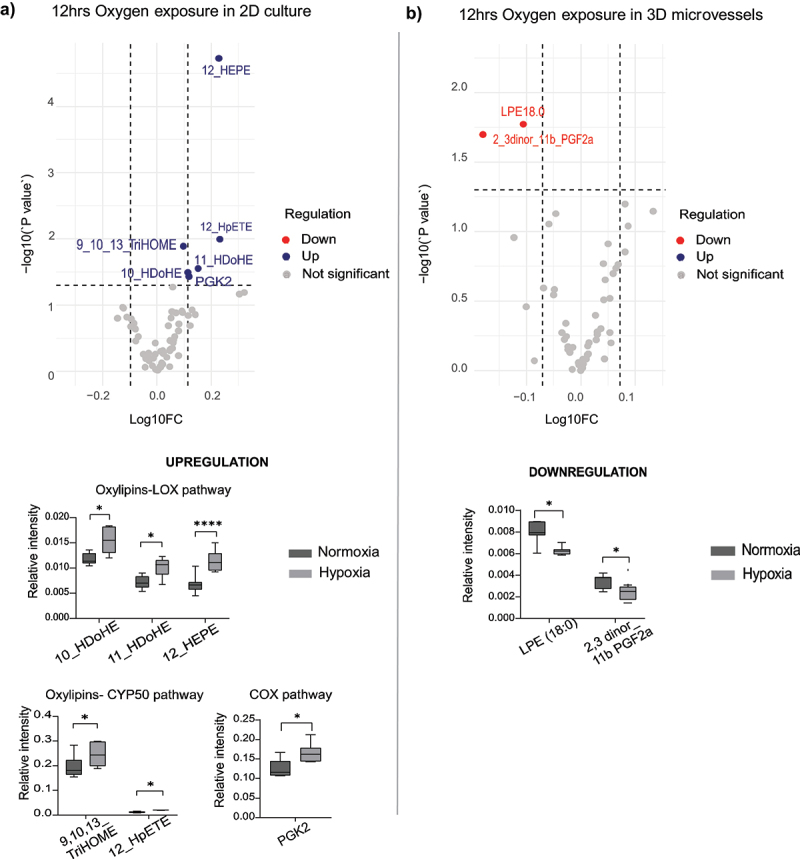
Measurement of signaling lipids in (a) 2D static culture and (b) 3D microvessels after transient oxygen exposure (12 hours), n = 7. Significance determined by the students *T*- test. ns = not significant; *p < 0.05; **p < 0.01; ***p < 0.001; ****p < 0.0001.

Next, we compared the impact of hypoxia on the generation of a selected set of signaling lipids by the HCAECs between our static 2D and perfused 3D model. Conditioned media samples were collected for UPLCMS/MS analysis. In particular, we assessed prostaglandins, lysosphingolipids, eicosanoids, and oxylipins. The control samples are the cells exposed to normoxia (21% oxygen) for 12 hours and 48 hours. We conducted statistical analysis on metabolites that passed the QC threshold during the assessment of the bioactive lipid composition. As shown in Figure 3, transient hypoxia exposure to HCAECs in a static 2D culture displayed increased levels of oxylipins from CYP450 pathway such as trihydroxyoctadecenoic acid (TriHOME) and hydroperoxyeicosatetranoic acid (HpETE). Also increased were oxylipins from the LOX pathway (hydroxydocosahexanoic acid (HDoHE) and hydroxy eicosapentanic acid (HEPE) and from the COX-2 metabolite Prostaglandin K2 (PGK2) (Figure 3(a)).

In the perfused 3D vasculature model (prostaglandin F2a) PGF2a and lysophosphatidylethanolamine (LPE) were downregulated (Figure 3(b)). The upregulation of oxylipins indicates that the hypoxic exposure increased the generation of reactive oxygen species by the HCAECs and the upregulation of all these enzymes in hypoxic conditions were shown in earlier studies.^27,42,43^

**Figure 4.**
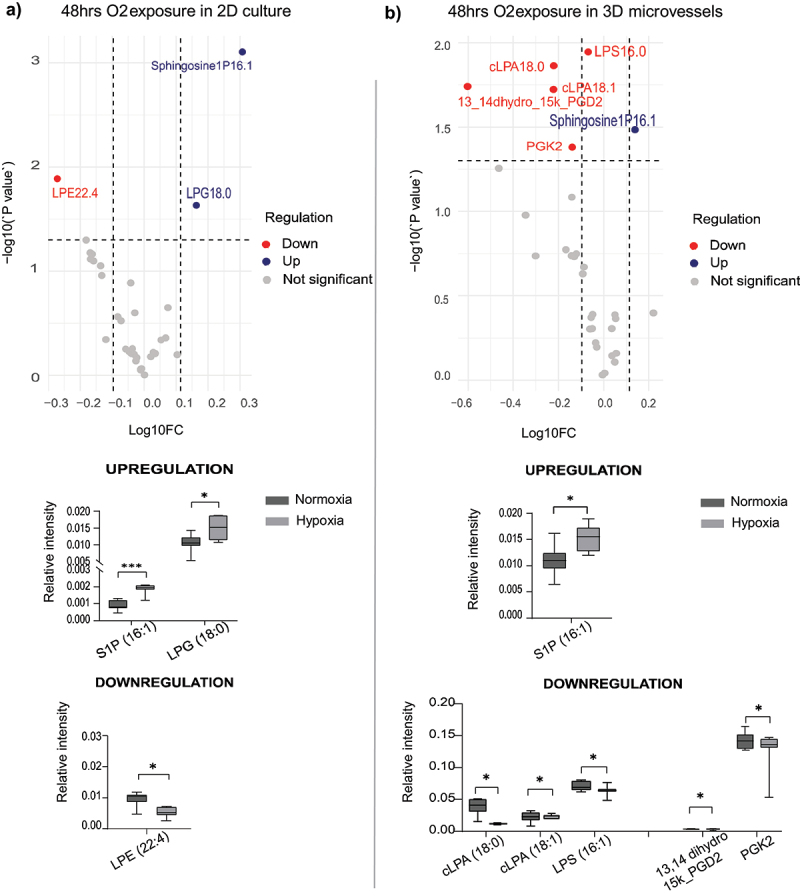
Measurement of signaling lipids in (a) 2D static culture and (b) 3D microvessels after persistent oxygen exposure (48 hours), *n* = 7. Significance is determined by the students T-test. ns = not significant; *p < 0.05; **p < 0.01; ***p < 0.001; ****p < 0.0001.

48 hrs of persistent hypoxic exposure of the HCAECs in the static 2D cultures revealed the upregulation of Sphingosine1P (S1P) and lysophosphatidylglycerol (LPG) and the downregulation of LPE and lysophosphatidic acid (LPA) (Figure 4(a)). The increased generation of Sphingosine1P and fatty acids was also seen in the perfused 3D model. Persistent 48 hr hypoxia decreased the levels of cyclic lysophosphatidic acid (cLPA), lipopolysaccharide (LPS), prostaglandin D2 (PGD2) and PGK2 in the conditioned media in this model (Figure 4(b)).

## 4. Discussion

Perfused in vitro 3D microfluidic vascular models are increasingly used to study human microvasculature. Compared to the 2D monolayer cultures, these microfluidic models are thought to provide more physiological responses due to their configuration and exposure to various degrees of shear stress. Here, we set out to compare both models in their response to hypoxia, focusing on two main homeostatic signaling pathways. Both models responded to 48 hrs of hypoxia as evidenced by the accumulation of HIF-1α in the nuclei of the HCAECs, consistent with an earlier study demonstration a time- and ROS dependent stabilization of HIF-1α in response to an environment of 1% oxygen. However, in the perfused 3D microvessels only 50% of the nuclei stained positive for HIF-1α. A likely explanation could be that the increased expression of the shear stress induced kruppel-like factor 2 in the HCAECs in the 3D model could inhibit stabilization of HIF-1α.^44^

While most previous studies reported hypoxia-mediated downregulation of eNOS activity,^15,17,34–36^ opposite results were also reported, and the differences may well be explained by the different models used.^22^ We recently developed a tracer-based methodology that is sufficiently sensitive and reproducible to assess eNOS activity in our small-scale microfluidic 3D microvascular model.^38^ Clearly, we observed no differences between normoxia and hypoxia both in the static 2D model and the 3D perfused model. However, when assessing the ratios of L-citrulline over L-ornithine in the 3D model, unlike in the 2D model we observed a lowering of the relative eNOS activity over that of arginase after 48 hrs of hypoxia (supplementary figure S2). A potential explanation would be a hypoxia-induced increase of arginase II activity as has been reported in a study with human lung endothelial cells.^45^ However, in the absence of an elevation of the absolute level of L-ornithine (Figure 2(b)), reduced eNOS activity due to the 48 hs of hypoxia would be the most likely explanation for this change in the L-citrulline/L-ornithine ratio.

Numerous signaling lipids can facilitate the endothelial response to changing environmental cues or inflammation.^45–49^ In a previous study we optimized the methodology for the profiling of bioactive lipids in our 3D microvessels model. We demonstrated that, in response to the pro-inflammatory cytokine TNF- α, static 2D cultures release a more inflammatory repertoire of bioactive lipids compared to perfused 3D-microvascular models.^50^ Also, the activating, anti-inflammatory impact of shear stress on membrane phospholipid metabolism,^51^ and the vasodilatory compound prostacyclin (PGI_2_) were reported previously.^7,52^ Consistent with these studies, in the static 2D culture model we observed a short-term hypoxia related increase in the levels of oxidative stress markers such as HDoHE, HEPE, TriHome, and HpETE potentially resulting from the peroxidation of their substrates.^53^ Given the fact that we observed that the translocation of HIF-1α is not established after the 12 hrs of hypoxia in this model, the increased release of these markers of oxidative stress is most likely unrelated to the action of HIF-1α.

The modulation of endothelial functions in hypoxic conditions is influenced by arachidonic acid-derived prostaglandin.^54^ Under shear stress conditions, a significant downregulation of prostaglandins (PGF2α) and (PGD2) is observed, partially associated with hypoxia, although not statistically significant in 2D culture hypoxic conditions. The reduced levels of PGF2α are linked to anti-inflammatory properties, and its higher levels reported in inflammation and atherosclerosis.^55–57^ Research indicates that higher shear stress increases PGD2 synthesis, while lower shear stress (below 5 dyne/cm^2^) shows no effects.^58^ Our observations suggest that the lower levels of PGF2α may be associated with shear stress, as HIF-1 α was not observed after 12 hours of hypoxia. Similarly, the lower level of PGD2 under persistent hypoxic conditions could be attributed to a combination of shear stress and hypoxia, consistent with the 50% HIF-1α translocation. However, the combined impact of hypoxia and shear stress on prostaglandin synthesis remains unclear.

The significance of lysophospholipids as a crucial lipid mediator in inflammatory conditions has been acknowledged.^59^ Earlier investigations involved studying the impact of LPAs on endothelial dysfunction in HCAECs through dosage treatment. This resulted in a decrease in eNOS activation alongside elevated levels of superoxide anion and ROS.^60^ In contrast, a few other studies have highlighted the association between LPAs and their modulation of endothelial cell functions, including cell proliferation, migration, barrier function, and capillary-like tube formation.^48,61–64^ Our study, conducted in a 3D model under prolonged hypoxia, revealed diminished levels of LPAs (cLPA 18:0, 18:1), possibly influenced by shear stress-mediated regulation. The reduced levels in 3D microvessels align with the observation that laminar flow decreases toll-like receptor 2 (TLR2) expression, a gene crucial in endothelial inflammation triggered by LPS induction.^65,66^ This suggests that, in our model, flow plays a role in downregulating inflammatory compounds.

S1P, a potent and versatile phospholipid, plays a crucial role in eliciting diverse vascular cell responses such as migration, proliferation, and survival. Additionally, it is implicated in pathological events related to endothelial dysfunction, including atherosclerosis, thrombosis, and inflammation.^67–70^ The signaling mechanism of S1P under hypoxic conditions has been previously elucidated,^71^ where the accumulation of S1P in endothelial cells, facilitated by HIF-1α and HIF-2α, results in an increased level of sphingosine kinase (SK) protein.^72^ In our study, exposure of microvessels to short-term hypoxia showed no significant changes in S1P, while prolonged exposure led to a significant upregulation of S1P levels. This suggests a regulatory mechanism through HIFα that induces S1P levels. Notably, the impact of hypoxia on S1P levels influences the vascular model, as very brief hypoxia (30 minutes) increases S1P protein expression in coronary artery endothelial cells and induces vasodilation.^73^ Given that S1P upregulation was observed in both 2D and 3D culture platforms, it strongly indicates a HIF-1a-mediated regulatory mechanism.

## 5. Conclusion

Examining hypoxia in endothelial cells is vital for understanding the adaptive mechanisms and its substantial involvement in various physiological and pathological processes. Importantly, oxygen-dependent pathways such as NO and signaling lipid pathways play a crucial role in vascular homeostasis and resolving inflammation in specific contexts. Our research highlights the significant impact of low oxygen levels on the metabolism of nitric oxide and signaling lipids, particularly in a 3D microvessels model. The exploration of the combined effects of hypoxia and shear stress in an in vitro culture setup is an underexplored area. Our study suggests that our model may offer relevance and appropriateness for analyzing metabolite levels under both normoxic and hypoxic conditions.

Collectively, our findings indicate that exposure to hypoxia in a 2D static culture environment leads to an increase in oxidative stress metabolites, a response mediated by the HIF-1α mechanism. Conversely, the hypoxic 3D platform demonstrates flow-mediated regulation of eNOS-based NO metabolites (L-citrulline+ and L-ornithine +7) and bioactive lipids. These changes contribute to anti-inflammatory effects. Immunostaining analysis of HIF-1α was consistent with our findings. Our study unveils a novel mechanism governing HIF-1α and flow-mediated regulatory metabolisms, proposing new therapeutic strategies for addressing arterial endothelial dysfunctions in prolonged hypoxic conditions.

## Supplementary Material

Supplemental Material

## Data Availability

The data that support the findings of this study are available in the Materials and Methods, Results, and Supplementary Material of this article. Additional data will be provided upon request.
