# Supplementary material for: Metabolic alterations of endothelial cells under transient and persistent hypoxia: study using a 3D microvessels-on-chip model
Source: Tissue Barriers. 2024 Nov 25;13(3):2431416. doi: 10.1080/21688370.2024.2431416 (PMC12506904; doi:10.1080/21688370.2024.2431416)
Supplement: Supplemental Material [file KTIB_A_2431416_SM5061.docx]

**Supplementarty figures**

**Suplementary figure 1**

**Suplementary figure 2**

**Suplementary figure 3**

**Suplementary Table 1**

| Metabolites name | Mass (Q1) | Mass (Q3) |
| --- | --- | --- |
| L-arginine | 345.170 | 171.000 |
| L-arginine [M+10] | 355.170 | 171.000 |
| L-citrulline | 346.150 | 171.000 |
| L-citrulline [M+9] | 355.150 | 171.000 |
| L-ornithine | 237.100 | 171.000 |
| L-ornithine [M+7] | 240.600 | 171.000 |

**Supplementary files**

**Supplementary material 1**

# Design and culture method of the 3D micro vessel model

HCAECs were cultured in Endothelial Cell Growth Medium-2 (PromoCell C-39216). We used a modified 2-lane OrganoPlate (MIMETAS) with pump-compatible inlets and outlets for microvessel culture. Phase guides separated the microvascular and ECM channels. The ECM channels were loaded with neutralized 4 mg/ml rat tail collagen type I (Trevigen 3440-005-01) neutralized using 10% each of Na2CO3 and HEPES buffer. After 10 minutes of polymerization at 37°C and 5% CO2, observation windows were filled with 50 µL HBSS+ to maintain optical clarity and prevent gel desiccation. For cell seeding, each microvascular channel inlet received 20 µL of 1% gelatin solution. After 30 minutes, this was replaced with 20 µL of media containing cells at 15 x 10^6 cells/mL. Following a one-hour incubation (37°C, 5% CO2) for microvessels formation, 50 mL of culture media was added to the channel inlets and outlets. Continuous bidirectional flow was established using a perfusion rocker (7° angle, 8-minute cycle). Media was refreshed after 48 hours, followed by unidirectional perfusion for 24, 48, and 96 hours before hypoxic experiments.

**Supplementary figure captions**

**Figure.1.** **Measurement of real time oxygen in a 3D micro vessel-on-a-chip model.** (A) Percentage of oxygen in normoxic condition (21% oxygen) and (B) Percentage of oxygen in hypoxia (1% oxygen). Control – Chip with no cells seeded, Microvessels – HCAECs seeded. Measurement was performed in LABVIEW software.

**Figure.2. Representation of our 3D micro vessel-on-chip model and the 2D model.**

Illustration of re-routed OrganoPlate and its (B) Single chip design was explained below. Every microfluidic chip structure is positioned underneath 4 adjacent wells and it consists of two channels: 1) an ‘perfusion’ channel (pink color) and 2) a ‘gel’ channel (ECM) (blue color). Every first well (1) and fourth well (4) is positioned on top of the inlet and outlet of the perfusion channel, while every second well (2) are for the gel channel. And every third well (3) is used for imaging and observation of the experiment. (C) Perfusion and gel channel was shown vertical and horizontal view and its separated by a phase guide (*). (D) Immunostaining of HCAECs in a chip. After 48 hours of cell seeding, a confluent vessel of HCAECs were immunostained with V-Cadherin and DAPI. (E) Image of a microfluidic pump with the two metal tubes attached to the inlet and outlet of rerouted OrganoPlate to carry fluid and create unidirectional flow. (F) Representation of 2D steady state culture model and (G) Immunosttaining of HCAECs in a 2D platform. Green – F-actin; Blue- Hoeschst. Scale bar – 50 µm.

**Figure.3. Measurement of isotope-labeled nitric oxide (NO) metabolites ratio** Citrulline+9/Arginine+10, Ornithine+7/Arginine+10, and Citrulline+9/Ornithine+7 in 2D static culture and in 3D microvessels-on-chip model under transient and persistent hypoxic conditions. Significance is determined by the one-way ANOVA multiple comparison test. ns = not significant; *, P<0.05; **, P<0.01; ***, P <0.001; ****, P<0.0001; #, P<0.0001.

**Supplementary Table.1**. MRM method and the compound list with target m/z for parent and product ions for tracer metabolites measurement.
